# Supplementary material for: Patterns of Intron Gain and Loss in Fungi
Source: PLoS Biol. 2004 Nov 30;2(12):e422. doi: 10.1371/journal.pbio.0020422 (PMC532390; doi:10.1371/journal.pbio.0020422)
Supplement: Table S1 — Also available at http://genes.mit.edu/NielsenEtAl/. (4.3 MB ZIP). [file pbio.0020422.st001.zip › NielsenEtAl/html/1421.html]

AN2110.1.NCU01057.1.MG10254.1.FG02691.1


```
 CLUSTAL W (1.82) Multiple Sequence Alignments - Introns Inserted


Sequence 1: NCU01057.1	409 aa
Sequence 2: FG02691.1	400 aa
Sequence 3: MG10254.1	409 aa
Sequence 4: AN2110.1	399 aa
Alignment Length: 409 aa
Number Identitical Residues: 232 aa
Alignment Score (without introns) 11398


MG10254.1 	MAPGALISEPPSAINIVTKRPSSTTTPSNNQADRSIFPDGIRTSGQHNPIYSLLRPYQDF
NCU01057.1	MAPGILVEEAQSTADASPSVFKHKNAYRNQDAPRDIFPDGIRTSGQHPPLYDVLKPYAEF
FG02691.1 	MAPGALIDESSHITLP---------QAKSTEAPRSIFPDGIRTSGQHPPLYNALKPYSEF
AN2110.1  	MAT--LTQTSAPAPAP--------AQIIHSNAPRDIFPDGLKTTGQHPPIYEELHPFEDF
          	**.  * . .                    :* *.*****::*:*** *:*. *:*: :*

MG10254.1 	PKSITGPTVWQKEQFEADATSWTHRFTPSEVAELGATADAFIASGVDLTGISK~SNFPLP
NCU01057.1	PKEIAGPTVWKREDFVNNPERWVHPFTDEEVQELSDTADAFIASGTPLTGISQ~ENFPLP
FG02691.1 	PTEITGPTLWKKEDYESKPERWTHPFTDEEIQELSDTADAFIASGTPLTGISK0SNFPLP
AN2110.1  	PKSIEGRTLWKAEDYKDAPEKWTHRFSAEEVEELGATADAFLASGTPLTGISK0SNFPLP
          	*..* * *:*: *::   .  *.* *: .*: **. *****:***. *****: .*****

MG10254.1 	TLHRTLEALRADLLDGKGFVLFKGFPATEWGPRKTATAYMGLGTHLGYFVSQNGRGHVLG
NCU01057.1	KLGTVLTNLRDDLLNGKGFILFKRFPADVWGAEKNAVAYMGLGTYLGYFLSQNGRGHVLG
FG02691.1 	KLGKVLETLREDLLNGKGFILFKRFPADVWGPLKNAVAYMGLGTYIGYFVSQNGLGHVLG
AN2110.1  	KLSALLYELRDDLLNGKGFILFKGFPVQEWGNHKSAVAYMGLGTYLGYFVSQNSRGHVLG
          	.*   *  ** ***:****:*** **.  **  *.*.*******::***:***. *****

MG10254.1 	HVKDVGDDAGQIHRVRIYRTAAR~QFFHADDGDLVGLLCVHRAAEGGESDLVSVHAVWNE
NCU01057.1	HVKDVGDDPTQIHTVRIYRTTAR~QFFHADDGDIVGLLCVHRAQEGGESDIVSVHHVWNT
FG02691.1 	HVKDVGDDATQIDKVRIYRTTAR~QFFHADDSDIVGLLCVHRAREGGESDIVSIHNVWND
AN2110.1  	HVKDLGEDPTQIDSVRIYRTNAR2QYFHADDSDIVGLLCIARALEGGESDIVSTHHVYNT
          	****:*:*. **. ****** ** *:*****.*:*****: ** ******:** * *:* 

MG10254.1 	LQQRHPDVAELLTRPIWYFDRKGEVSEGQEEWTRQPVFYLEPGGKGRVYCK~FNPYYVRS
NCU01057.1	LQQEHPDVAELLTKPIWYFDRKGEVSEGQQEWVRQPVVYLENGGKGRLYCK~WDPYYVKS
FG02691.1 	LQKNNPDVAELLTKPVWYFDRKGETSVGEEEWIRTSIFYLENGGQGRVYCK~WDPYFVRS
AN2110.1  	LAAERPDVLKTLIEPIWYFDRKGETSKGQEEYIRTSVIYLERGDNPRVYTK2WDPYYVRS
          	*  ..*** : * .*:********.* *::*: * .:.*** *.: *:* * ::**:*:*

MG10254.1 	LTRFSDKGIIPPLSEEQKRAIEILEDTCQRLALHMILEVGDIQFLSNAHILHARTAYKD1
NCU01057.1	LTRFSDKGLIPALSEEQLRAMQILEETCQRLALHMILEVGDIQFLSNAHLLHARTAYKD1
FG02691.1 	LTRFSDKGIIPALSEEQKRAITILEETCQKLALHMVLEVGDIQFVSNTHLLHARTAYTD1
AN2110.1  	LTRFSDAGLIPPLSDRQIEALEVLEQTCQRLSLHMILEVGDIQFVSNSHVLHARTAYKD~
          	****** *:**.**:.* .*: :**:***:*:***:********:**:*:*******.* 

MG10254.1 	FPPPAPRRHLLRLWLSTPDNEGGWVLPFPDSGEKKRGGIQVNNTPPRCPLDAE
NCU01057.1	FAPPAPRRHLLRLWLATPESEGGWALPMPDSDEKKRGGIQVNNTPPKAPLDAE
FG02691.1 	HNPPTPRRHLLRLWLSTPETEGGWALPFHDSKEKKRGGIQVNNNAPRCPLDAE
AN2110.1  	YAPPAPRRHLMRLWLSTPESEGGWRLPFWDSNEKKRGGVQVDDTPPVALLDAE
          	. **:*****:****:**:.**** **: ** ******:**::..* . ****
```
